# Pulsed Radiofrequency of the Trigeminal Ganglion for Treating Postherpetic Neuralgia of the Ophthalmic Branch

**DOI:** 10.1155/2021/6638392

**Published:** 2021-05-30

**Authors:** Dong-Yang Liu, Jin-Sheng Chen, Ze-Zang Fang, Shao-Yan Liu, Li Wan

**Affiliations:** Department of Pain Management, The State Key Clinical Specialty for Pain Medicine, The Second Affiliated Hospital, Guangzhou Medical University, Guangzhou, Guangdong 510260, China

## Abstract

Postherpetic neuralgia (PHN) is a painful, long-lasting condition as a consequence of nerve damage resulting from a herpes zoster infection. Although there are many different treatments available to reduce pain duration and severity, PHN is often refractory to them and no single therapy shows an effective cure for all cases of PHN, especially for those involving the ophthalmic branch of the trigeminal nerve. Pulsed radiofrequency (PRF) is a minimally invasive procedure for pain treatment that has been practiced over the past decade. However, its clinical efficacy and safety for treating PHN involving the ophthalmic branch of the trigeminal nerve have not been evaluated. *Objective*. This study aimed to evaluate the efficacy and safety of PRF for treating PHN involving the ophthalmic branch of the trigeminal ganglion. *Study Design*. An observational study. *Setting*. All patients received PRF of the ophthalmic branch of the trigeminal nerve, pain intensity was assessed by a visual analogue scale (VAS), and complications before and after PRF stimulation were noted. *Methods*. Thirty-two patients with PHN of the ophthalmic branch were treated by PRF of the ophthalmic branch with controlled temperature at 42°C for 8 min. Pain relief, corneal reflex, sleep quality, and satisfaction were assessed for all patients. *Results*. Thirty out of 32 patients (93.75%) reported significant pain reduction after PRF treatment. Twenty-eight of them (87.5%) were satisfied with their sleep and obtained a pain score lower than 3 following the procedure. Only two patients had a recurrence of the severe burning pain and returned to the hospital for other medical therapies 2 weeks after the PRF procedure. No patient lost the corneal reflex. *Limitations*. This study is an observational study and a nonprospective trial with a short-term follow-up period. *Conclusion*. PRF of the trigeminal ganglion of the ophthalmic branch can significantly reduce pain sensation and improve sleep quality and satisfaction for PHN of the ophthalmic branch.

## 1. Introduction

People infected with the varicella-zoster virus are at risk of developing herpes zoster. Although most cases resolve spontaneously, the pain associated with herpes zoster does not resolve in a substantial number of patients, resulting in a chronic pain condition called postherpetic neuralgia (PHN). PHN is the most common complication of zoster infection and remains a challenging condition to treat. It has been reported that an estimated 12.5% of patients with zoster infection aged ≥50 years develop PHN three months after zoster infection onset and the proportion affected increases sharply with age [1]. Among adults, herpes zoster infects the trigeminal nerve in 15–20% of the cases, with the ophthalmic division being most affected [2]. Following herpes zoster infection of the ophthalmic branch, ocular complications associated with poor visual outcomes include acute corneal lesions, retinitis, optic neuritis, and uveitis. Besides these ocular complications, patients may also develop PHN [3]. Of the symptoms, ophthalmic PHN is the most painful symptom and is characterized by severe burning and lancinating pain often associated with allodynia.

Medication, nerve blocks, and chemical neurolytic blocks have been used to treat PHN of the ophthalmic branch. Unfortunately, the effects are limited and often produce intolerable side effects. In recent years, spinal cord stimulation has been found to be a useful technique for the treatment of intractable chronic neuropathic pain [4]; however, the neuralgia from the ophthalmic branch could hardly be controlled by stimulation in the spinal cord [5, 6]. Peripheral nerve stimulation could also be used to treat herpetic neuralgia and especially more effectively in acute and subacute phases [7, 8]. Pulsed radiofrequency (PRF) is a method that has been used in chronic pain therapy for several decades and has been developed widely in clinical practice [9]. For this therapy, a radiofrequency current is generated intermittently, and heat is washed out during a silent period, which causes minor tissue injury surrounding the needle puncture and prevents nerve degeneration [10]. We herein summarize the efficacy of PRF in the ophthalmic branch to treat intractable PHN, suggesting that PRF is a possible treatment for ophthalmic branch neuralgia.

## 2. Materials and Methods

### 2.1. Methods

#### 2.1.1. Patients

Forty-eight patients with PHN of the ophthalmic branch were recruited between August 2014 and February 2017 from the Department of Pain Management, the Second Affiliated Hospital of Guangzhou Medical University. Inclusion criteria were diagnosis of classic PHN of the ophthalmic branch. Patients experienced lancinating or burning pain, paresthesia, or pruritus for over three months. All patients reported moderate to most intense pain (>5) on a visual analogue score (VAS), ranging from 0 (no pain) to 10 (the most intense pain). Ten patients had facial numbness, decreased corneal reflex, or visual impairment on the symptomatic side. Anticonvulsants such as pregabalin 75 mg q12 h were used to treat lancinating pain, while tramadol 100 mg q12 h and a tricyclic antidepressant drug amitriptyline 12.5 mg qn were prescribed for easing burning pain. Patients were excluded from the study if they fulfilled one of the following criteria: noncompliance with physician's advice, infection on the skin or the deep tissue at the puncture site, the presence of bleeding tendencies, or receiving anticoagulant therapy which could not be replaced with intravenous low-molecular-weight heparin subcutaneous injection. In addition, patients with unstable, severe cardiovascular or cerebrovascular disease, such as trigeminal neuralgia secondary to cranial tumors, were also excluded. Sixteen patients were excluded based on these criteria. This study was approved by the Institute Review Board of the Second Affiliated Hospital of Guangzhou Medical University. Informed consent for participation in this study was obtained from the patients before the treatment.

#### 2.1.2. Surgical Procedure

**Figure 1:**
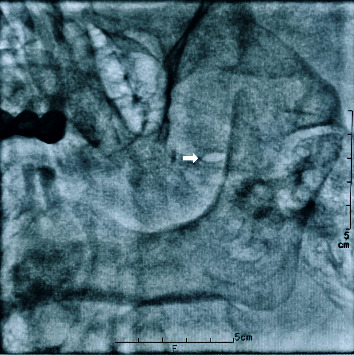
The image showed the oval foramen, located at the upper third of the mandibular ramus, inside of the condyle, penetrating the ophthalmic branch of the trigeminal ganglion toward the medial corner of the oval foramen (arrow).

**Figure 2:**
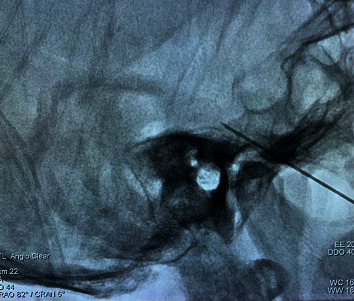
The position of the ophthalmic branch of the trigeminal ganglion is located over the slope line 3 mm and the trocar tip was shown in the right position.

Our technique was carried out as previously described [10]. The patient was placed in a supine position with the head extended on the Digital Subtraction Angiography (DSA) bed. Standard American Society of Anesthesiology monitors were utilized throughout the procedure. Each patient was premedicated with an intravenous (i.v.) injection of 0.5 mg atropine to maintain the heart rate over 90 and sufentanil 0.08 *μ*g/kg i.v was administered by bolus. Following sterile prep and drape, the C-arm was positioned 15–25 degrees ipsilaterally and 30–35 degrees caudally to show the foramen ovale, located at the upper third of the mandibular ramus, inside of the condyle (Figure 1). A 3 ml of 1% lidocaine was infiltrated in the subcutaneous tissues and a 10 cm long radiofrequency needle with a diameter of 0.7 mm and a 2 mm active tip was directed toward the foramen ovale. The needle trajectory was adjusted fluoroscopically until the radiofrequency trocar resided in proximity to the foramen ovale. A bolus of 1 mg/kg propofol was administered intravenously before the trocar penetrated the foramen ovale to avoid the penetrating pain to the trigeminal ganglion. The final location of the final needle tip was positioned over the slope line 3 mm, as shown in Figure 2; then, the patient was awakened to give the sensorial and motor stimulation. A tissue impedance was controlled around 200–300 Ώ. Motor stimulation 2 Hz with 1.5 mV was performed to exclude motor twitch. Sensorial stimulation 50 Hz with 0.1–0.3 mV was performed to induce paresthesia in the area of the ophthalmic division. After sensorial and motor stimulation, pulsed RF (PRF) was administered with a radiofrequency generator (COSMAN Radiofrequency Therapy Apparatus, USA) at a pulse width of 20 ms and a controlled temperature of 42°C for 8 minutes. We selected a temperature around 42°C to avoid damage to neural structures and tested the corneal reflex, pain sensation, and numbness after PRF. No patients had a loss of the corneal reflex. No additional anesthetics were administered during the PRF treatment. After PRF treatment, the patients continued to use the following medications in the follow-up period: gabapentin 0.1, tid; amitriptyline 12.5 mg, qn; tramadol 50 mg, q12 h.

#### 2.1.3. Observations and Follow-Up

VAS: baseline VAS was recorded prior to the procedure, immediately following the PRF, and at 1 week, 1 month, and 3 months. The effect of PRF was evaluated by assessing pain relief immediately after the procedure. Scores were divided into the following categories: 0 for no pain, from 1 to 3 for mild pain, 4 to 6 for moderate pain, and 7 to 10 for the worst possible pain.

The pain treatment effect (VAS reduction) was divided into the following four grades, the number of patients in each grade was counted: grade1, pain score less than 2; grade 2, pain score 3 or more on the VAS scale; grade 3, pain score 6 or more on the VAS scale; grade 4: complete relief. The number of patients in each grade was counted separately.

Sleep quality was divided into the following five levels evaluated by the patients themselves: 1, good sleep; 2, relatively satisfied sleep; 3, sleep after medicine; 4, poor sleep; 5, cannot sleep. The number of patients with different sleep quality levels was counted before and 1 month after PRF.

The patient's treatment satisfaction was divided into the following five grades: 1, strongly dissatisfied; 2, somewhat dissatisfied; 3, neutral; 4, somewhat satisfied; 5, very satisfied.

#### 2.1.4. Statistics

Statistical analyses were performed using Prism 5.0 software. The immediate postoperative VAS was analyzed using the chi-square test. We used a linear mixed model with a Toeplitz covariance structure (smallest Akaike information criterion) for the analysis of repeated measures structure; an analysis of the primary and secondary endpoints of the full analysis set, which contained unbalanced data, was conducted. A *p* value < 0.05 was deemed statistically significant and *p* value < 0.001 was deemed statistically very significant.

## 3. Results

After 16 patients were excluded based on the exclusion criteria, data from 32 patients with PHN of the ophthalmic branch (20 males and 12 females aged 56–86 years) were analyzed in the study. The duration of disease ranged from 0.5 years to 3 years, with an average of 1.5 ± 0.75 years. The patients' detailed information is shown in Table 1. All patients had no nerve block such as supratrochlear, supraorbital, and stellate ganglion before ophthalmic nerve PRF.

**Figure 3:**
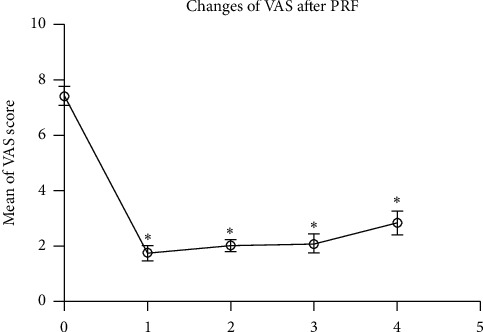
The VAS score was significantly decreased after PRF for 1d (1) and follow-up at 1 month (2), 2 months, (3) and 3 months (4) when compared to pre-PRF baseline (0).^*∗*^*p* < 0.001 indicates pre-PRF vs. post-PRF.

Pain was efficiently reduced in all patients after the PRF during follow-up. The number of patients in grade 3 pain relief increased gradually to 30 after three months, while the number in grade 4 was 8 in the first week and dropped to 0 at the end of follow-up (Figure 3, Table 2). Only 2 of the 32 patients (6.25%) experienced mild pain relief (grade 2) of the skin in the forehead following the treatment and returned to medical therapy with oral tramadol 150 mg q12 h and amitriptyline 25 mg, bid. The other 30 patients' VAS were under 3 till the end of follow-up (Table 3).

For all patients, sleep quality significantly improved after the PRF procedure, in which 87.5% (28 of 32 patients) were satisfied with their sleep and obtained a pain score lower than 3 following the procedure (Table 4).

Twenty-eight patients (68.75%) were satisfied after the PRF treatment during the follow-up period (Table 5).

## 4. Discussion

Following PRF of the ophthalmic branch of the trigeminal ganglion, intolerable pain, sleep quality, and quality of life were significantly improved in nearly all PHN patients. PHN is a classic neuropathic pain that presents as lancinating and burning pain and is associated with paresthesia. The paresthesia can be expressed as allodynia and hyperalgesia [3], where the patient is affected by the pain and also has to endure fatigue, insomnia, and reduced social activities [11, 12]. In our study, the patients' pain intensity was significantly decreased after the PRF treatment compared to that prior to the treatment. Studies have shown that patients who have received antiviral agents within 72 h of onset of the zoster rash experience reported significantly lower pain ratings than those who go untreated. However, if herpes zoster infection induces nerve damage that develops to chronic neuropathic pain, the patient can be affected by long-term excruciating pain and sleep disturbances. In our study, most of the patients also showed sleep disturbances before the PRF procedure and reported remarkable improvement after the treatment for at least 3 months. This suggests that the PRF treatment of the ophthalmic branch is maybe an option for the treatment of ophthalmic branch neuralgia.

Studies have shown that mortality and severity of PHN are closely related to the patient's age, the severity of the rash, prodromal pain symptom, and gender [13]. The incidence of PHN increases with age [13–15]. In our study, the age of PHN was between 58 and 86, with an average of 69 years. These data are consistent with previous reports [15]. It has been reported that 56% of patients had thoracic dermatomes affected, and up to 25% of patients had cranial trigeminal nerve involvement, most commonly of the first division [16, 17]. Herpes zoster ophthalmicus (HZO) is 20 times more common when compared with either mandibular or maxillary infection [18], being surpassed only by thoracic zoster [19, 20]. There are multiple pharmacological treatments determined by pain characteristics, such as anticonvulsants that block the lancinating pain [3] and tricyclic antidepressants prescribed for burning pain. Nerve blocks with steroid injections and physical therapy with low- or high-frequency electric stimulation have also been used to treat PHN [21–23]; however, the effect is limited, especially in patients with neuralgia for the ophthalmic branch of the trigeminal nerve. As a result, patients must take a higher dose of analgesics, often with significant adverse effects.

Nerve stimulation is another method to control neuropathic pain via both central and peripheral pathways [3, 24–26]; however, the high cost, limited effect, and discomfort when applying electrodes on the face on the trigeminal branch 1 area increased the preference for other alternative methods for treating neuralgia of V1 PHN. Deep brain stimulation into the thalamus nuclei is another effective invasive technique [3]; however, the patient must undergo a craniotomy. Because of their invasiveness in brain, motor cortex stimulation and deep brain stimulation are the last resort therapies for postherpetic ophthalmic neuralgia.

Thermal radiofrequency has been used for treating trigeminal neuralgia for decades and the ophthalmic branch was not a contraindication [27]. Previously, physicians have applied radiofrequency coagulation to the affected nerve branch; however, the pain symptoms disappeared only temporarily and were replaced with new forms of pain, similar to insect bites or ants crawling on the skin [6]. In recent decades, physicians have tried PRF to treat PHN patients and have received good results [9]. Our clinical practice has confirmed previous reports with a short-term follow-up period. Kim et al. [28] have reported that pain intensity was significantly reduced and last for 12 weeks after the dorsal root ganglion PRF in PHN patients. Our observation is consistent with the previous study and showed even further results in the trigeminal ganglion of the ophthalmic branch of PHN; that is, the PRF can significantly reduce patient's pain sensation immediately and its effect can last for at least 3 months after the procedure. Our findings suggest that PRF is a useful method to treat PHN of the trigeminal ophthalmic branch.

Previous research showed that PRF delivered at 42°C on the rat dorsal root ganglion will not induce structural changes aside from transient endoneurial edema and collagen deposition [29]. More recent studies have shown recovery of upregulated inflammatory cytokines on day 30 after PRF, mild axonal damage, and little swelling of the mitochondria, which may lead to temporary blockage of nerve signals through the nerve pathway [30]. This little structural change may be related to the immediate effect of PRF and needs to be researched further. It is uncertain if this is the predominant mechanism underlying the efficacy of PRF. Because PHN is an intractable disease, a highly efficacious vaccine is a promising method to prevent HZ and PHN. It is encouraging that the new herpes zoster subunit vaccine has achieved excellent effect and might decrease the incidence rate of PHN [31]. However, if the patient suffers from PHN of the ophthalmic branch, the PRF of the trigeminal ganglion is an alternative method in addition to nerve stimulation.

## 5. Conclusion

In summary, PRF of the trigeminal ganglion of the ophthalmic branch can significantly reduce pain sensation and improve sleep quality and the quality of life; therefore, it may be a candidate for PHN of the ophthalmic branch.

## Figures and Tables

**Table 1:** Basic characteristics of patients for PRF to treat PHN of the trigeminal ophthalmic branch.

Parameter	(*n* = 32)
Age (years, mean ± SD)	69 ± 15
Gender (female/male)	12/20
Duration of symptoms (year, mean ± SD)	1.50 ± 0.75

*Division of trigeminal nerve, n (%)*	
V1	30 (94.8%)
V2	0 (0%)
V3	2 (5.2%)

**Table 2:** The number of patients in each grade at different time points after PRF (*n* = 32).

VAS	Follow-up period		
	1 week	1 month	3 months
Grade 1 (pain score less than 2)	0	0	0
Grade 2 (pain score 3 or more)	2	2	2
Grade 3 (pain score 6 or more)	22	26	30
Grade 4 (complete relief)	8	4	0

**Table 3:** Follow-up period and VAS scores (*n* = 32).

VAS	Presurgery	Follow-up period			
		1 day	1 week	1 month	3 months
0–3 (no pain to mild pain)	0	30^*∗*^	30^*∗*^	30	30
≥4 (moderate to worst pain)	32	2^*∗*^	2^*∗*^	2	2

^*∗*^ *p* < 0.001, pre-PRF treatment compared to follow-up period for 1 day, 1 week, 1 month, and 3 months.

**Table 4:** The number of patients with different sleep quality levels, pre- and post-PRF (*n* = 32).

	Good sleep	Relatively satisfied sleep	Sleep after medicine	Poor sleep	Cannot sleep
Pre-PRF	0	6^*∗*^	9	15	2
Post-PRF	4	24	4	0	0

^*∗*^ *p* < 0.001, pre-PRF surgery compared to follow-up period for 1 month.

**Table 5:** The patient's satisfaction after PRF (*n* = 32).

	Very satisfied	Somewhat satisfied	Neutral	Somewhat dissatisfied	Dissatisfied
Case	7	15	6	2	2
%	21.87	46.88	18.75	6.25	6.25

## Data Availability

The data used to support the findings of this study are included within the article.
