# Corrigendum to “Pulsed Radiofrequency of the Trigeminal Ganglion for Treating Postherpetic Neuralgia of the Ophthalmic Branch”

**DOI:** 10.1155/2021/9791801

**Published:** 2021-12-14

**Authors:** Dong-Yang Liu, Jin-Sheng Chen, Ze-Zang Fang, Shao-Yan Liu, Li Wan

**Affiliations:** Department of Pain Management, The State Key Clinical Specialty for Pain Medicine, The Second Affiliated Hospital, Guangzhou Medical University, Guangzhou, Guangdong 510260, China

In the article titled “Pulsed Radiofrequency of the Trigeminal Ganglion for Treating Postherpetic Neuralgia of the Ophthalmic Branch” [1], the acknowledgement section should be corrected as follows:

“The authors thank Chong-Rong Gao and Zhen-He Lu for their initial help with the project and members of the Department of Pain Management for their comments. Moreover, special thanks are due to Li Hu for the advice on this manuscript. The research was supported by the National Natural Science Foundation of China (No. 81771182) to Li Wan and by the High-Level University Construction Promotion Project of Guangzhou Medical University (2017-No. 160-9) to Li Wan.”
